# Serum urea acid and urea nitrogen levels are risk factors for maternal and fetal outcomes of pregnancy: a retrospective cohort study

**DOI:** 10.1186/s12978-022-01496-6

**Published:** 2022-09-15

**Authors:** Lanlan Wu, Yao Liu, Zengyou Liu, Hengying Chen, Siwen Shen, Yuanhuan Wei, Ruifang Sun, Guifang Deng

**Affiliations:** 1 Department of Clinical Nutrition, Union Shenzhen Hospital of Huazhong University of Science and Technology, No. 89 Taoyuan Road, Shenzhen, 518052 Guangdong People’s Republic of China; 2 Department of Obstetrics, Union Shenzhen Hospital Huazhong University of Science and Technology, Shenzhen, People’s Republic of China; 3 Injury Prevention Research Center, Shantou University Medical College, Shantou, People’s Republic of China

**Keywords:** Urea acid, Urea nitrogen, Adverse pregnancy outcomes, Small for gestational age infants

## Abstract

**Background:**

In recent years, results on the association between serum uric acid (UA) and pregnancy outcomes have been inconsistent, and the association between urea nitrogen (UN) and adverse pregnancy outcomes in normal pregnant women has not been reported. Thus, we examined the association of UA and UN levels during gestation with the risk of adverse pregnancy outcomes in a relatively large population.

**Methods:**

A total of 1602 singleton mothers from Union Shenzhen Hospital of Huazhong University of Science and Technology at January 2015 to December 2018 were included. Both UA and UN levels were collected and measured during the second (16–18th week) and third (28–30th week) trimesters of gestation respectively. Statistical analysis was performed using multivariate logistic regression.

**Results:**

After adjustment, the highest quartile of UA in the third trimester increased the risk of premature rupture of membranes (PROM) and small for gestational age infants (SGA) by 48% (odds ratio [OR]: 1.48, 95% confidence interval [CI]: 1.04–2.10) and 99% (95% CI: 1.01–3.89) compared to those in the lowest quartile. The adjusted OR (95% CI) in the highest quartile of UN for the risk of SGA was 2.18 (95% CI: 1.16–4.13) and 2.29 (95% CI: 1.20–4.36) in the second and third trimester, respectively. In the second trimester, when UA and UN levels were both in the highest quartile, the adjusted OR (95% CI) for the risk of SGA was 2.51 (95% CI: 1.23–5.10). In the third trimester, when the group 1 (both indicators are in the first quartile) was compared, the adjusted ORs (95% CI) for the risk of SGA were 1.98 (95% CI: 1.22–3.23) and 2.31 (95% CI: 1.16–4.61) for group 2 (UA or UN is in the second or third quartile) and group 3 (both indicators are in the fourth quartile), respectively.

**Conclusions:**

Higher UA and UN levels increased the risk of maternal and fetal outcomes. The simultaneous elevation of UA and UN levels was a high-risk factors for the development of SGA, regardless of whether they were in the second or third trimester.

**Supplementary Information:**

The online version contains supplementary material available at 10.1186/s12978-022-01496-6.

## Plain language summary

Adverse pregnancy outcomes are important public health problems in terms of high mortality and long-term health effects of maternal and newborn babies. This study assessed the association between serum urea acid and urea nitrogen levels during pregnancy and the risk of adverse pregnancy outcomes in Chinese women. The study was conducted between January 2015 and December 2018. Serum uric acid and urea nitrogen were measured at weeks 16–18 and 28–30, respectively. A total of 1602 singleton pregnant women participated in the study. We found that elevated levels of uric acid and urea nitrogen increased the risk of maternal and infant outcomes. In addition, we found for the first time that elevated uric acid and urea nitrogen concentrations were a risk factor for SGA, both in the second and third trimesters. Therefore, monitoring maternal uric acid and urea nitrogen biochemical parameters during pregnancy is necessary to optimize nursing and intervention. Furthermore, uric acid and urea nitrogen are simple, inexpensive, and readily available tests and should be evaluated additionally.

**Supplementary Information:**

The online version contains supplementary material available at 10.1186/s12978-022-01496-6.

## Background

Adverse pregnancy outcomes such as premature rupture of membranes (PROM), premature birth (PTB), low birth weight infants (LBW), and small for gestational age infants (SGA) not only threaten the health of pregnant women and fetuses in the perinatal and postpartum period but also affect the long-term health of infants by increasing the risks of neonatal infections, infectious disease, and growth faltering [1–3]. Thus, accurate identification and management of risk factors for adverse pregnancy outcomes are highly desirable for optimizing care and interventions.

The occurrence and development of adverse pregnancy outcomes are closely related to the status of maternal health during pregnancy, such as levels of serum uric acid (UA) and urea nitrogen (UN). UA is the end product of purine catabolism, which acts as an antioxidant and reduces DNA damage at physiological concentration. However, a high concentration of UA constitutes a risk factor for diseases such as gout, hypertension, and cardiovascular disease, as it could promote inflammation and endothelial dysfunction [4, 5]. High levels of maternal UA can spread to the placenta, enter the fetal circulation, induce placental inflammation and dysfunction, and ultimately prevent fetal development [6]. Hyperuricemia has been used as a diagnostic marker of preeclampsia, and has been widely used to monitor the severity of hypertensive disease during pregnancy [7, 8]. In recent years, many studies have investigated the correlation between UA and pregnancy outcomes, however, the findings have been inconsistent. An elevated serum UA level has been previously reported to be an independent risk factor for adverse pregnancy outcomes such as PTB, LBW, and SGA by some studies [9–13], but not by others [14]. In addition, the sample size of these population-level studies was relatively small, and the association between UA and a wider range of other pregnancy outcomes was not studied.

UN is the main end product of protein metabolism and is another indicator of kidney function. Elevated concentrations of UN have been demonstrated to promote the production of reactive oxygen species in mouse models [15]. Recent in vitro experiments have confirmed that a high concentration of urea itself can lead to endothelial dysfunction and activation of the proatherosclerotic pathway [16]. Several case reports investigated the association between blood indexes and the pregnancy outcome of pregnant women undergoing hemodialysis and found that UN was negatively correlated with birth weight and gestational age, and that a low UN level was conducive to optimizing the pregnancy outcomes in these women [17–20]. However, the association between the UN and adverse pregnancy outcomes in normal pregnant women has not yet been reported.

In the present study, we aimed to examine the association of UA and UN in the second and third trimesters of pregnancy with adverse pregnancy outcomes including PROM, PTB, LBW, and SGA, and to evaluate the influence of the combination of the two indicators in the second and third trimester of pregnancy on maternal and infant complications in a retrospective cohort study.

## Methods

### Study design and participants

This retrospective cohort study was conducted at Union Shenzhen Hospital of Huazhong University of Science and Technology (Shenzhen, Guangdong Province, China) from January 2015 to December 2018. A total of 1716 pregnant Chinese women who registered and attended for their routine first hospital visit in pregnancy at the Antenatal Department were enrolled in the present study. The exclusion criteria were as follows: smoking or drinking alcohol during pregnancy (n = 3), history of liver disease (n = 10), diabetes or hypertension (n = 65), kidney disease (n = 4), heart disease (n = 3), and twin or multiple pregnancy (n = 29). Finally, a total of 1602 gravidas with singleton pregnancies were included in the present study. Baseline demographic information and medical history of the participants (Additional file 1: Supplementary Table1) were collected at the beginning via a structured interview, and UA and UN were measured in the second (16–18th) and third (28–30th) trimester of gestation, respectively. In addition, the participants were followed up until delivery. Pregnancy outcomes were recorded in the hospital information system. The study was approved by the Ethics Committee of the Union Shenzhen Hospital of Huazhong University of Science and Technology and conducted in accordance with the Declaration of Helsinki as set forth by the World Medical Association.

### Data collection and outcome definition

Age (years), education (primary, secondary, and college or above), conception method (natural or artificial), gravidity, parity (primiparity or multiparity)**,** history of miscarriage (yes or no), embryo number and history of diseases (e.g., liver disease, diabetes or hypertension, kidney disease, and heart disease) were obtained through face–to–face interviews by a well-trained investigator and questionnaires were completed simultaneously. The height and weight of each participant were measured with an accuracy of 0.1 cm and 0.1 kg, respectively, using an electronic scale with participants wearing light clothing and no shoes. Pre-pregnancy body mass index, BMI (kg/m^2^) was calculated by dividing the weight (kg) by the square of the height (m^2^).

The definition of adverse pregnancy outcomes was followed the definition of the International Classification of Diseases, 10th Revision (ICD–10). PROM was defined when the membranes were observed to have ruptured before the onset of labor [21–23]. PTB was defined as delivery at ≥ 20 weeks and before completing 37 weeks of gestation. LBW was defined as a newborn with a birth weight of less than 2500 g. SGA babies usually have birth weights below the 10^th^ percentile for babies of the same gestational age on the growth chart [24].

### Laboratory assays

Fasting venous blood samples were collected by a professionally trained investigator at 16–18th weeks and 28–30th weeks of pregnancy. The samples were centrifuged at 3500 rpm for 5 min at 4 °C within 2 h of collection. The UA and UN levels were measured by enzymatic assay. All laboratory measurements were performed using an ACCELERATOR a3600 automatic analyzer (Abbott, Chicago, USA).

### Statistical analyses

Baseline information was presented as means (SD) for continuous variables and proportion (%) for categorical variables. UA and UN were categorized by quartile distribution with the first quartile serving as the reference. Odds ratios (ORs) and 95% confidence intervals (95% CIs) were calculated by using logistic regression models for examining the association of UA and UN during gestation with the risk of adverse pregnancy outcomes across each of the quartiles. Logistic regression models were run for the major adverse outcomes after adjusting confounding factors, and two models were included in the present study: Model 1 was not adjusted, Model 2 was adjusted for age, pre-pregnancy BMI, education, conception method, number of pregnancies, parity, history of miscarriage, gestational diabetes mellitus (GDM), and gestational hypertension. The data were regrouped based on the quartiles of UA and UN as follows: Group1—both indicators are in the first quartile, Group2—UA or UN is in the second or third quartile, and Group3—both indicators are in the third quartile as well as the above two models (Model 1 and Model 2). All analyses were carried out by using SPSS 24.0 software (SPSS Inc., Chicago, IL, USA), wherein a two–sided *p*-value of < 0.05 was considered statistically significant. Graphic production was completed by using R version 3.0.3 software (The R Foundation for Statistical Computing, Vienna, Austria).

## Results

### Baseline characteristics

A total of 1602 singleton pregnant women aged 31.58 (± 3.82) years were included in the study, having a BMI of 20.78 ± 3.31 kg/m^2^ BMI and having 38.91 ± 1.19 weeks of gestational age, on average, at the time of delivery. Among those, 1320 (82.4%) women had a college education or above, 1582 (98.8%) women conceived naturally, 906 (56.6%) women were multiparity, and 682 (42.6%) women had a history of miscarriage. The average number of gravidity was 2.23 ± 1.15. The differences in UA and UN between the second trimester and third trimester were statistically significant (*p* < 0.05), and the levels of UA and UN in the third trimester were higher than those in the second trimester. As presented in Table 1, 313 cases of PROM, 37 cases of PTB, 29 cases of LBW, and 83 cases of SGA were included in this study.

**Table 1:** Baseline characteristics of all pregnant women in this study

Characteristics of maternal and neonatal	
No. of maternal	1602
Age (years)	31.58 ± 3.82
Age categories	
< 35	1217 (76.0)
≥ 35	385 (24.0)
Pre-pregnancy BMI (kg/m^2^)	20.78 (3.31)
Education, n (%)	
Primary	54 (3.4)
Secondary	228 (14.2)
College or above	1320 (82.4)
Conception method	
Natural	1582 (98.8)
Artificial	11 (0.7)
Number of pregnancies	2.23 ± 1.15
Parity, n (%)	
Primiparity	696 (43.4)
Multiparity	906 (56.6)
History of miscarriage, n (%)	682 (42.6)
Gestational age at delivery (weeks)	38.91 ± 1.19
UA (μmol/L)	
Second trimester	209.88 ± 43.57
Third trimester	286.04 ± 64.00
UN (mmol/L)	
Second trimester	2.61 ± 0.63
Third trimester	2.94 ± 0.73
Blood pressure (mmHg)	
SBP	117.10 ± 11.91
DBP	66.62 ± 9.38
OGTT	
FPG (mmol/L)	4.60 ± 0.34
1 h (mmol/L)	7.99 ± 1.63
2 h (mmol/L)	6.98 ± 1.32
Birth weight (kg)	3322.55 ± 410.83
Adverse maternal and fetal outcomes, n (%)	
GDM	310 (19.4)
Gestational hypertension	44 (2.7)
PROM	313 (19.5)
PTB	37 (2.3)
LBW	29 (1.8)
SGA	83 (5.2)

Data was presented as mean (SD) for continuous variables and n (%) for categorical. *BMI* body mass index, *UA* urea acid, *UN* urea nitrogen, *SBP* systolic blood pressure, *DBP* diastolic blood pressure, *OGTT* oral glucose tolerance tests, *FPG* fasting plasma glucose, *GDM* gestational diabetes mellitus, *PROM* premature rupture of membranes, *PTB* premature birth, *LBW* low birth weight infants, *SGA* small for gestational age infants

### Association of UA and UN with adverse pregnancy outcomes

Table 2 shown the ORs (95% CIs) for adverse pregnancy outcomes considering the UA levels. After adjusting for confounding factors, no significant relationship was found between UA levels and the risk of PROM, PTB, LBW, and SGA in the second trimester, whereas a dose–response relationship was found between the UA levels and the risk of PROM and SGA. Women with UA levels in the fourth quartile had a 48% (OR = 1.48, 95% CI: 1.04–2.10) (*P*_trend_ = 0.022) and 99% (OR = 1.99, 95% CI: 1.01–3.89) (*P*_trend_ = 0.066) higher risk of PROM and SGA, respectively, than those in the first quartile. For every one standard deviation (SD) increase in UA concentrations, there was a 20% (OR = 1.20, 95% CI: 1.07–1.36) and 24% (OR = 1.24, 95% CI: 1.01–1.53) increase in the risk of PROM and SGA, respectively.

**Table 2:** ORs (95%CI) for the adverse pregnancy outcomes according to the quartiles of urea acid (UA)

	UA (μmol/L) in the second trimester				*P *_*trend*_	Per–SD increase
	Q1 (< 179.8)	Q2 (179.8–205.4)	Q3 (205.5–234.7)	Q4 (> 234.7)		
PROM						
Case/N	76/401	83/401	75/400	79/400		
Model 1	1 (ref)	1.16 (0.79, 1.58)	0.99 (0.69, 1.41)	1.05 (0.74, 1.49)	0.960	1.01 (0.89, 1.14)
Model 2	1 (ref)	1.10 (0.77, 1.57)	1.01 (0.70, 1.44)	1.02 (0.71, 1.46)	0.821	1.01 (0.88, 1.14)
PTB						
Case/N	5/401	10/401	9/400	13/400		
Model 1	1 (ref)	2.03 (0.69, 5.98)	1.82 (0.61, 5.49)	2.66 (0.94, 7.53)	0.089	1.27 (0.94, 1.72)
Model 2	1 (ref)	2.02 (0.67, 6.04)	1.85 (0.61, 5.65)	2.70 (0.94, 7.80)	0.095	1.27 (0.94, 1.73)
LBW						
Case/N	7/401	8/401	6/400	8/400		
Model 1	1 (ref)	1.15 (0.41, 3.19)	0.86 (0.29, 2.57)	1.15 (0.41, 3.20)	0.928	1.01 (0.70, 1.46)
Model 2	1 (ref)	1.19 (0.42, 3.42)	0.99 (0.32, 3.08)	1.35 (0.47, 3.89)	0.669	1.09 (0.75, 1.61)
SGA						
Case/N	20/401	16/401	23/400	24/400		
Model 1	1 (ref)	0.79 (0.40, 1.55)	1.16 (0.63, 2.15)	1.22 (0.66, 2.24)	0.334	1.14 (0.92, 1.41)
Model 2	1 (ref)	0.87 (0.44, 1.72)	1.36 (0.73, 2.56)	1.47 (0.78, 2.75)	0.123	1.22 (0.98, 1.51)

	UA (μmol/L) in the third trimester					
	Q1 (< 240.1)	Q2 (240.2–277.7)	Q3 (277.8–323.0)	Q4 (> 323.0)		
PROM						
Case/N	69/407	74/394	76/406	94/395		
Model 1	1 (ref)	1.13 (0.79, 1.63)	1.12 (0.79, 1.62)	1.53 (1.08, 2.17)	0.022	1.20 (1.07, 1.36)
Model 2	1 (ref)	1.15 (0.80, 1.66)	1.10 (0.76, 1.58)	1.48 (1.04, 2.10)	0.047	1.18 (1.04, 1.33)
PTB						
Case/N	13/407	7/394	7/406	10/395		
Model 1	1 (ref)	0.55 (0.22, 1.39)	0.53 (0.21, 1.35)	0.79 (0.34, 1.82)	0.531	1.11 (0.81, 1.53)
Model 2	1 (ref)	0.55 (0.21, 1.40)	0.56 (0.22, 1.43)	0.79 (0.34, 1.84)	0.565	1.12 (0.82, 1.53)
LBW						
Case/N	11/407	4/394	8/406	6/395		
Model 1	1 (ref)	0.36 (0.12, 1.17)	0.72 (0.29, 1.82)	0.56 (0.20, 1.52)	0.378	0.99 (0.68, 1.43)
Model 2	1 (ref)	0.35 (0.11, 1.12)	0.71 (0.28, 1.84)	0.52 (0.19, 1.45)	0.338	0.97 (0.67, 1.41)
SGA						
Case/N	14/407	23/394	19/406	27/395		
Model 1	1 (ref)	1.74 (0.88, 3.43)	1.38 (0.68, 2.79)	2.06 (1.06, 3.99)	0.069	1.28 (1.04, 1.57)
Model 2	1 (ref)	1.76 (0.89, 3.49)	1.42 (0.69, 2.87)	1.99 (1.01, 3.89)	0.066	1.24 (1.01, 1.53)

*PROM* premature rupture of membranes, *PTB* premature birth, *LBW* low birth weight infants, *SGA* small for gestational age infants. Model 1: without adjustment. Model 2: adjustment for age, pre–pregnancy BMI, education, conception method, number of pregnancies, parity, history of miscarriage, gestational diabetes mellitus and gestational hypertension

Table 3 shown the ORs (95% CIs) for adverse pregnancy outcomes considering the UN levels. A dose–response relationship was observed between the UN and the risk of SGA. The multivariable–adjusted ORs (95% CIs) for the highest quartile of UN levels as compared to the lowest quartile were 2.18 (95% CI: 1.16–4.13) (*P*_trend_ = 0.007) and 2.29 (95% CI: 1.20–4.36) (*P*_trend_ = 0.002) in the second and third trimesters, respectively. For every one SD increase in the UN levels, the value leaned toward 21% (OR = 1.21, 95% CI: 0.98, 1.50) in the second trimester and 35% (OR = 1.35, 95% CI: 1.11, 1.64) in the third trimester.

**Table 3:** ORs (95%CI) for the adverse pregnancy outcomes according to the quartiles of urea nitrogen (UN)

	UN (mmol/L) in the second trimester				*P*_*trend*_	Per–SD increase
	Q1 (< 2.23)	Q2 (2.23–2.50)	Q3 (2.55–3.00)	Q4 (> 3.00)		
PROM						
Case/N	88/458	76/352	80/442	69/350		
Model 1	1 (ref)	1.16 (0.82, 1.63)	0.93 (0.66, 1.30)	1.03 (0.73, 1.47)	0.838	0.97 (0.86, 1.10)
Model 2	1 (ref)	1.15(0.82, 1.63)	0.95(0.68, 1.34)	1.05(0.73, 1.50)	0.938	0.98(0.86, 1.11)
PTB						
Case/N	9/458	7/352	12/442	9/350		
Model 1	1 (ref)	1.01 (0.37, 2.75)	1.39 (0.58, 3.34)	1.32 (0.52, 3.35)	0.440	1.02 (0.73, 1.40)
Model 2	1 (ref)	0.98 (0.35, 2.71)	1.36 (0.56, 3.32)	1.26 (0.48, 3.28)	0.500	0.98 (0.71, 1.37)
LBW						
Case/N	8/458	4/352	8/442	9/350		
Model 1	1 (ref)	0.65 (0.19, 2.17)	1.04 (0.39, 2.79)	1.49 (0.58, 3.89)	0.349	1.10 (0.77, 1.56)
Model 2	1 (ref)	0.75(0.22, 2.58)	1.03(0.38, 2.85)	1.58(0.58, 4.29)	0.346	1.12(0.78, 1.61)
SGA						
Case/N	18/458	12/352	27/442	26/350		
Model 1	1 (ref)	0.86 (0.41, 1.82)	1.59 (0.86, 2.93)	1.96 (1.06, 3.64)	0.011	1.18 (0.96, 1.46)
Model 2	1 (ref)	0.90(0.43, 1.92)	1.54(0.83, 2.87)	2.18 (1.16, 4.13)	0.007	1.21(0.98, 1.50)

	UN (mmol/L) in the third trimester					
	Q1 (< 2.45)	Q2 (2.45–2.90)	Q3 (2.93–3.35)	Q4 (> 3.35)		
PROM						
Case/N	87/408	85/455	55/339	86/400		
Model 1	1 (ref)	0.85 (0.61, 1.18)	0.72 (0.49, 1.04)	1.01 (0.72, 1.41)	0.874	1.01 (0.89, 1.14)
Model 2	1 (ref)	0.83 (0.59, 1.16)	0.65 (0.44, 0.96)	0.91 (0.65, 1.29)	0.443	0.97 (0.85, 1.10)
PTB						
Case/N	14/408	8/455	6/339	9/400		
Model 1	1 (ref)	0.50 (0.21, 1.21)	0.51 (0.19, 1.33)	0.65 (0.28, 1.51)	0.309	0.74 (0.52, 1.06)
Model 2	1 (ref)	0.50 (0.21, 1.22)	0.49 (0.18, 1.33)	0.68 (0.28, 1.60)	0.354	0.75 (0.52, 1.08)
LBW						
Case/N	8/408	8/455	4/339	9/400		
Model 1	1 (ref)	0.89 (0.33, 2.41)	0.60 (0.18, 2.00)	1.15 (0.44, 3.01)	0.898	1.01 (0.69, 1.45)
Model 2	1 (ref)	0.82 (0.30, 2.27)	0.54 (0.15, 1.87)	1.06 (0.39, 2.86)	0.996	0.99 (0.68, 1.44)
SGA						
Case/N	15/408	16/455	20/339	32/400		
Model 1	1 (ref)	0.96 (0.47, 1.96)	1.64 (0.83, 3.26)	2.28 (1.21, 4.28)	0.002	1.38 (1.13, 1.68)
Model 2	1 (ref)	0.86 (0.42, 1.79)	1.57 (0.78, 3.16)	2.29 (1.20, 4.36)	0.002	1.35 (1.11,1.64)

*PROM* premature rupture of membranes, *PTB* premature birth, *LBW* low birth weight infants, *SGA* small for gestational age infants. Model 1: without adjustment. Model 2: adjustment for age, pre–pregnancy BMI, education, smoking status, alcohol status, conception method, number of pregnancies, parity, history of miscarriage, gestational diabetes mellitus and gestational hypertension

### Association of combined classification of UA and UN with adverse pregnancy outcomes

**Fig. 1:**
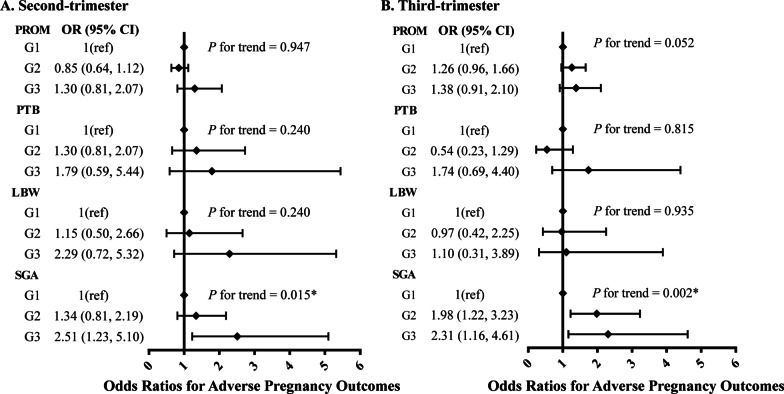
Pregnancy outcomes of pregnant women according to combination of UA and UN. The model was adjusted for age, prepregnancy BMI, education, conception method, number of pregnancies, parity, history of miscarriage, gestational diabetes mellitus and gestational hypertension

As seen in Fig. 1, women in the G3 group (both UA and UN are in the third quartile) in the second trimester had an increased risk of SGA, while it was found that the risk of SGA was increased in both G2 and G3 groups. In the second trimester, the multivariable–adjusted ORs (95% CIs) across the quartiles of the groups combined UA and UN were 1 (reference; G1), 1.34 (0.81–2.19; G2), and 2.51 (1.23–5.10; G3; *P*_trend_ = 0.015) (Fig. 1A). In the third trimester, the multivariable–adjusted ORs (95% CIs) across the quartiles of the groups combined UA and BUN were 1 (reference; G1), 1.98 (1.22–3.23; G2) and 2.31 (1.16–4.61; G3; *P*_trend_ = 0.002) (Fig. 1B). However, no significant relationship was observed between the groups and the risk of PROM, PTB, and LBW.

## Discussion

In this retrospective cohort study of Chinese women, we investigated the association of UA and UN with adverse pregnancy outcomes. High UA levels in the second trimester were not significantly associated with adverse pregnancy outcomes, but with an increased risk of PROM and SGA in the third trimester was observed. We also found that mothers with elevated UN had a higher risk of SGA, whether they were in the second or third trimester of pregnancy. Moreover, we found for the first time that pregnant women with concurrently elevated UA and UN concentrations had a higher risk of giving birth to SGA infants.

After entering the fetal circulation, high levels of UA affect fetal development by causing placental inflammation and dysfunction. In vitro studies have suggested that UA can induce inflammatory pathways in vitro, with activation of p38 MAPK, NF–κB, and AP-1 and an increased expression of COX-2 and MCP-1 [25]. Elevated of UA levels can also inhibit placental amino acid uptake, trophoblast invasion and the incorporation of trophoblast into endothelial monolayers, leading to placental hypoperfusion [26–28]. Additionally, during late gestation, UA crystals activate the nod-like receptor protein_3 (NLRP3) inflammatory pathwayvia an IL-1–dependent pathway, causing placental interface inflammation and affecting fetal development [29, 30]. Numerous population–level studies have also investigated the association between UA and adverse maternal and infant outcomes, although their conclusions have been inconsistent. In a retrospective analysis of 212 women in Pittsburgh, Laughon et al. attested that hyperuricemia in the second trimester (18–21 weeks of gestation) was associated with lower birthweight in normotensive women [10]. A prospective multicentric cohort study of 404 Iranian normotensive pregnant women indicated that maternal hyperuricemia in the third trimester (28–42 weeks of gestation) was independently associated with PTB (OR, 3.17; 95% CI, 2.1–4.79) and SGA (OR, 1.28; 95% CI, 1.04–2.57) [13]. Similar findings were also found in two studies: a case–control study carried out in 120 Japanese women in the third trimester with normal blood pressure by Akahori et al. [12] and a retrospective cohort study carried out in 1,880 Australian women by TL-A Hawkins et al. [11]. In contrast, in a prospective study that included 1,541 subjects, Laughon et al. indicated that elevated UA levels in the first trimester (less than 15 weeks of gestation) were not associated with PTB and SGA [14]. In the present study, we extended these findings to a relatively large cohort of Chinese pregnant women and observed that women in the fourth quartile of UA levels during the third trimester of pregnancy exhibit a 48% and 99% higher risk of PROM and SGA, respectively. During a normal pregnancy, the UA concentration changes dynamically. UA concentration is significantly reduced at 8 weeks of gestation, and these reduced levels remain stable until approximately 24 weeks of gestation, after which maternal UA levels increase rapidly to pre-pregnancy levels [31]. Heterogeneity in the results reported in previous studies may be due to variations in study design, sample size, the timing of biomarker evaluation, diagnostic criteria, or other confounding factors.

UN, which is generally recognized to be a biomarker of kidney function, is associated with pregnancy-induced hypertension such as preeclampsia. Previous experimental studies, both in vitro and in vivo, have indicated that urea-induced ROS stimulates activation of endothelial pro-inflammatory pathways by inhibiting glyceraldehyde-3-phosphate dehydrogenase (GAPDH), including increased protein kinase C isoforms activity, increased hexosamine pathway activity, and accumulation of intracellular advanced glycation end products (AGEs) [16]. Simultaneously, reactive oxygen species induced by urea also directly inactivated the antiatherosclerosis enzyme PGI2 synthase and also caused endoplasmic reticulum (ER) stress [32]. It is well established that the elevated blood UN level is an independent risk factor for adverse fetal pregnancy outcomes in pregnant women undergoing hemodialysis. Multiple case reports have shown that blood UN levels in pregnant women undergoing hemodialysis were negatively correlated with fetal birth weight and gestational age [17–20]. We initially explored the relationship between UN and adverse pregnancy outcomes in normal pregnant women, and observed that women in the fourth quartile of UN levels exhibited 118% and 129% higher risk of SGA during the second and third trimester of pregnancy, respectively. More animal studies and population epidemiological evidence are needed to elucidate the relationship between the UN and maternal and infant pregnancy outcomes.

To the best of our knowledge, this study is the first to assess the relationship between the combined association of UA and UN concentrations with the risk of developing adverse pregnancy outcomes. An interesting finding from our study was that women with higher levels of both UA and UN levels exhibited a 151% higher risk of SGA during the second trimester of pregnancy. Another striking finding was that women with both UA and UN levels in the second or third quartile had a 98% higher risk of developing SGA during the third trimester and in the fourth quartile had a 131% higher risk of developing SGA during the third trimester. These results highlighted the importance of paying attention to UA and UN concentrations across the whole duration of pregnancy. However, further longitudinal studies with larger sample sizes are needed to validate our findings.

Although our study comprehensively examined the relationship between maternal renal function indicators and the risk of adverse pregnancy outcomes using two parameters in a relatively large sample size, some limitations still exist. Firstly, the analytic cohort was from China, which may limit the generalizability of the study results. Secondly, although we accounted for known confounders, some unmeasured or unknown residual confounders remained (either unmeasured or unknown). Finally, the small size of the subgroup of women aged > 35 years and with a BMI of > 24 kg/m^2^ limited the statistical power. However, the chosen biochemical parameters of UA and UN to assess maternal renal function are simple, inexpensive, and readily available tests, and thus should be additionally evaluated.

## Conclusions

In summary, the present study demonstrated that higher UA and UN levels increased the risk of maternal and fetal outcomes. Simultaneously elevated UA and UN levels was a high-risk factor for the development of SGA, regardless of whether they were in the second or third trimester.

## Supplementary Information

**Additional file 1. Supplementary Table 1.** Timeline of information collection for pregnant women.

## Data Availability

The datasets used and/or analyzed during the current study are available from the corresponding author on reasonable request.

## Abbreviations

BMI: Body mass index
GAPDH: Glyceraldehyde-3-phosphate dehydrogenase
GDM: Gestational diabetes mellitus
NLRP3: Nod-like receptor protein3
ORs: Odds ratios
95% CIs: 95% Confidence intervals
PROM: Premature rupture of membranes
PTB: Premature birth
LBW: Low birth weight infants
SGA: Small for gestational age infants
SD: Standard deviation
UA: Urea acid
UN: Urea nitrogen
