# Supplementary material for: Serum urea acid and urea nitrogen levels are risk factors for maternal and fetal outcomes of pregnancy: a retrospective cohort study
Source: Reprod Health. 2022 Sep 15;19:192. doi: 10.1186/s12978-022-01496-6 (PMC9479307; doi:10.1186/s12978-022-01496-6)
Supplement: Supplementary file 1 — Additional file 1. Supplementary Table 1. Timeline of information collection for pregnant women. [file 12978_2022_1496_MOESM1_ESM.docx]

Supplementary Table1 Timeline of information collection for pregnant women.

| Information for pregnant women | Baseline | Second trimester | Third trimester | Delivery |
| --- | --- | --- | --- | --- |
| Demographic data |  |  |  |  |
| Age (years) | √ |  |  |  |
| Education (primary, secondary, and college or above) | √ |  |  |  |
| Height (m) | √ |  |  |  |
| Wight (kg) | √ |  |  |  |
| Smoking status (yes or no) | √ |  |  |  |
| Alcohol status (yes or no) | √ |  |  |  |
| Medical history |  |  |  |  |
| History of disease (yes or no) |  |  |  |  |
| Liver disease | √ |  |  |  |
| Diabetes | √ |  |  |  |
| Hypertension | √ |  |  |  |
| Kidney disease | √ |  |  |  |
| Heart disease | √ |  |  |  |
| Conception method (natural or artificial) | √ |  |  |  |
| Number of pregnancies | √ |  |  |  |
| Parity (primiparity or multiparity) | √ |  |  |  |
| Embryo number | √ |  |  |  |
| History of miscarriage (yes or no) | √ |  |  |  |
| Blood pressure (SBP/DBP, mmHg) | √ | √ | √ | √ |
| OGTT (FPG/1h/2h, mmol/L) |  | √ |  |  |
| UA (μmol/L) |  | √ | √ |  |
| UN (mmol/L) |  | √ | √ |  |
| Gestational age at delivery (weeks) |  |  |  | √ |
| Birth weight (kg) |  |  |  | √ |

Abbreviations: UA: urea acid; UN: urea nitrogen; SBP: systolic blood pressure; DBP: diastolic blood pressure; OGTT: oral glucose tolerance tests; FPG: Fasting plasma glucose.
